# Saw-box osteotomy versus reamer-box osteotomy in posterior stabilized total knee arthroplasty: a retrospective study of an average five year follow-up

**DOI:** 10.1007/s00264-024-06119-2

**Published:** 2024-02-22

**Authors:** Ahmed Abdelbadie, Ahmed A. Toreih, Moawed F. El-Adawy, Mohamed S. Arafa

**Affiliations:** 1 Department of Orthopedic Surgery and Trauma, Suez Canal University Hospital, Kilo 4.5 Ring Road, Ismailia, 41111 Egypt; 2 Department of Orthopedic Surgery, Fayoum University Hospital, Fayoum, Egypt

**Keywords:** Posterior stabilized, PFC Sigma, Genesis II, Femoral box osteotomy, Periprosthetic fractures, Reamer-box

## Abstract

**Purpose:**

The purpose of this study is to compare the difference of results between two methods of femoral box osteotomy adopted by two designs of posterior stabilized total knee prostheses.

**Patients and methods:**

Retrospective analysis of the results of two groups of patients operated upon using two primary PS TKA systems, PFC Sigma (DePuy Synthes, Johnson and Johnson®) and Genesis II prosthesis (Smith and Nephew®), with an average of five year follow-up was done. Group 1 included 152 knees in 121 patients and group 2 included 122 knees in 111 patients. The average follow-up period in both groups was five years. The box osteotomy method depends on bone saw in group 1, and bone reamer in group 2.

**Results:**

The KSS score of group 2 was better in the first six months postoperatively. Then, no significant differences were seen in the remaining follow-up visits. The risk of periprosthetic fractures was significantly higher in group 1 (*p*-value 0.040). Survival analysis showed a significantly shorter time for reoperation in group 1 than in group 2 as described by log-rank test, (*p* < 0.006).

**Conclusion:**

The method of box cutting has an impact on the function and longevity of posterior stabilized primary knee implants. The risk of periprosthetic fractures can be reduced by proper patient selection, decreasing the box sizes, and development of more “controlled” box osteotomy instruments.

## Introduction

Primary total knee arthroplasty (TKA) is an effective procedure to improve function and provide pain relief in patients with advanced osteoarthritis [1]. TKA was ranked as the second most common procedure performed in the USA in 2018, with a 134% increase from 2005 [2]. The two main designs of implants are the posterior cruciate retaining (CR) and the posterior cruciate substituting that is well known as the posterior stabilized (PS) design. PS implants attempt to replace the function of the PCL with a polyethylene (PE) post and femoral cam that prevent anterior translation of the femur on the tibia, while allowing femoral rollback during flexion [3]. The use of PS prostheses in TKA gained more popularity since the administration of the original Insall-Burstein prosthesis in 1978. Currently, its use increases in the USA in comparison to other TKA designs [4–6].

The superiority of PS designs over the CR designs or the vice-versa is still a matter of controversy. However, the PS TKA proved more feasibility of balancing severe coronal and sagittal deformities (i.e. varus, valgus, or recurvatum), more controlled flexion kinematics, less PE sliding wear, greater weight-bearing maximal flexion, and greater posterior femoral roll-back than are observed even with the more recent high-flexion CR TKA designs [7]. A lower patellofemoral contact pressure in PS TKA when compared with CR designs is another potential merit [8].

There are several potential disadvantages of PS implants with respect to other CR prostheses, and these include tibial post-wear or even breakage [9], increased incidence of patellar clunk syndrome with anterior knee pain [10], and flexion instability when the posterior offset is markedly reduced [11].

The main disadvantage of PS TKA design is being less bone-preserving. A big chunk of femoral bone attached to the PCL femoral insertion is resected to create a room to the PS mechanism. Although bone-preserving techniques are generally preferred by orthopaedic surgeons, the clinical consequences of the additional bone resection of the PS design are still under debate. Theoretically, this may lead to increase the risk of periprosthetic fractures and decrease the bone stock available for future revision surgeries. Despite these disadvantages, PS TKA remains the most used worldwide [12].

The purpose of this study is to compare the difference of results between two methods of box cutting, cutting by the saw adopted in PFC Sigma prosthesis (DePuy Synthes, Johnson and Johnson®) versus box cutting by reamer and chisel adopted in Genesis II prosthesis (Smith and Nephew®).

## Materials and methods

Retrospective analysis of the results of two groups of patients operated by the same surgeon by two primary PS TKA systems, PFC Sigma (DePuy Synthes, Johnson and Johnson®) and Genesis II prosthesis (Smith and Nephew®), with an average of five year follow-up was done. The demographic data of the patients including the preoperative co-morbidities are shown in (Table 1).

**Table 1:** Demographic data of the patients

Variable	Group 1 (152 knees)	Group 2 (122 knees)	*p*-value
Age (years)	68.2 ± 6.5, (55–81)	67.1 ± 6, (56–80)	0.151
Male/female	41/111	35/87	1.000
Duration of follow-up (years)	5.2 ± 2.1, (3.5–6.2)	5.0 ± 2, (3.2–5.6)	0.424
Body mass index	28.1 ± 2.5	27.8 ± 3.1	0.387
Preoperative co-morbidities* Hypertension Diabetes Heart disease Thyroid disease Chronic kidney disease BPH Spinal decompression Lupus erythematosus Previous cancer colon Previous breast cancer Post-phlebitic limb Depression	83 74 62 13 8 5 3 0 1 2 1 10	67 45 34 10 7 5 4 2 0 3 0 11	

^*^Heart disease includes patients with previous coronary bypass grafting; chronic kidney patients do not include patients with dialysis; previous spinal decompression patients do not include patients with radicular lower limb pain

Power analysis was carried out in advance and the study protocol was reviewed and approved by our institutional review board and ethical committee (#5486, November 2023). All patients provided their written informed consents to participate in this study.

### Eligibility criteria

Including criteria for the patients enrolled in the study include patients who were diagnosed with primary knee osteoarthritis of both sexes, age above 55, ability to walk prior to surgery, and indicated to PS TKR. Patients with a history of prior knee infection, rheumatoid arthritis, post-traumatic arthritis, previous osteotomy, failed uni-condylar prosthesis, vascular disease, or radicular pain were excluded. All surgeries were done under tourniquet with preoperative antibiotics and 1 gm of intravenous tranexamic acid was given prior to its inflation. Postoperative medications include three doses of intravenous antibiotics and prophylactic low molecular weight heparin (enoxaparin 40 mg) for two weeks postoperatively. Physiotherapy protocol starts on the first day postoperatively with full weight bearing on a walker frame, quadriceps training, and range of motion exercises.

### Operative procedure

All the patients were operated by the same surgical approach used in the two groups. After tourniquet inflation, the knee joint was exposed using the standard medial parapatellar approach. After everting the patella, distal femoral resection was done utilizing an intramedullary guide. The rotational alignment of the distal femur was determined based on the femur’s intercondylar axis, and femoral sizing, cuts, and chamfers were completed using an anterior reference system. Then, the proximal tibia was resected using an extramedullary alignment guide device aiming to get the tibial inclination perpendicular to the tibial mechanical axis in the coronal plane and the posterior tibial slope angle of 3° in the sagittal plane. Gap balancing was obtained by applying the principles of the modified gap technique. Patella was not resurfaced in all patients; it was only trimmed and denervated by cautery.

The first group included the patients operated by PFC Sigma prosthesis (152 knees in 121 patients). Box cutting in this group involves the application of a cutting jig to the distal femur and then removing the box piece by a saw in three cuts, distal, medial and lateral. This way is considered a traditional method that is adopted by many prosthetic manufacturers.

**Fig. 1:**
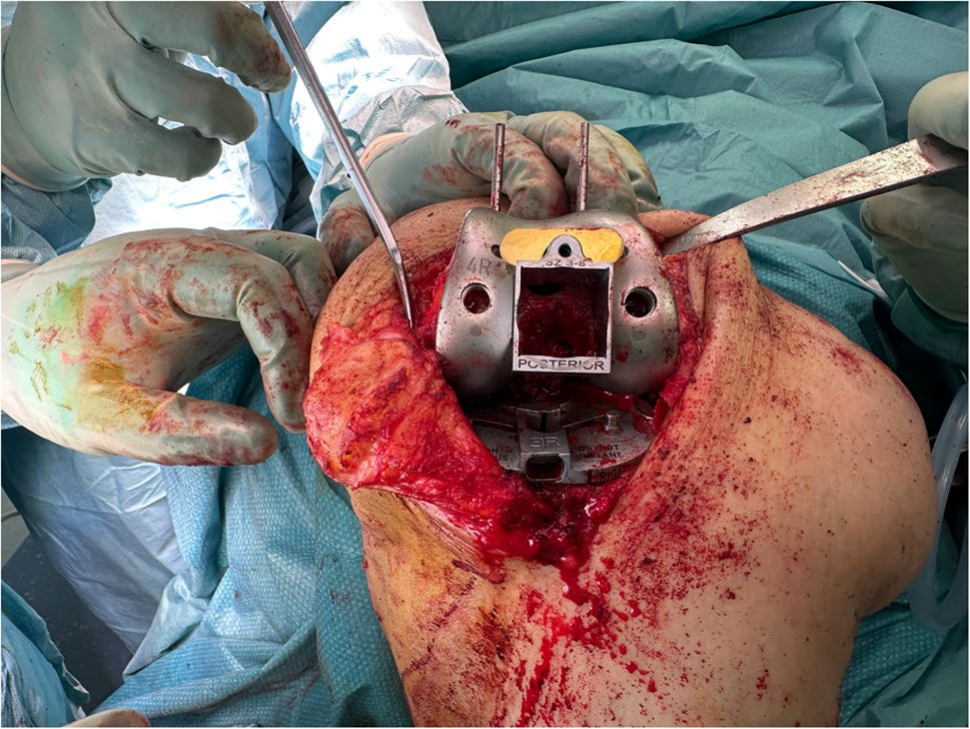
Genesis II prosthesis box osteotomy. This unique method of femoral box osteotomy depends on a square resection collet applied to the CR trial femoral component. The osteotomy is obtained by reaming through it before cutting the box with square chisel

The second group operated by Genesis II prosthesis (122 knees in 111 patients). Box cutting in this group utilizes the application of the square cutting jig (housing resection collet) on CR trial prosthesis and reaming the distal femoral box and cutting the box by a square chisel through the cutting jig (Fig. 1). This method of femoral box osteotomy is unique and not used in other implant designs.

Cementless trial prosthesis was then applied, and patellar tracking was tested by the no-thumb manoeuvre. Then, a pulse lavage was used to obtain a bloodless field without debris. Bone cement was then applied on both the implant and the bone surface. After inserting the PE trial implant, the knee joint was fully extended and maintained for the remaining of the total 15 min of cement preparation and cure time. After hardening of the cement, the excess cement was removed by a small osteotome and a hammer. Then, the tourniquet was released, and haemostasis was attempted. The tourniquet was inflated again, and the true PE fixed bearing insert was implanted after a second pulse lavage. Topical tranexamic (2 gm) was injected and no drain is inserted with closure.

The choice of the prosthesis done for each patient was random and dependent on the stock availability at the time of surgery. Bilateral cases were operated at two stages using the same design in each patient.

### Statistical analysis

The results of both groups were compared regarding operative data, including operative time, and intraoperative complications. Postoperative complications were registered, and the calculated knee society score KSS [13] was registered at six month follow-up and then yearly for an average of five years. New X-rays were taken on each follow-up visit. Seventeen patients were dropped from the follow-up, ten from the first group, and seven from the second group.

Data management was performed using SAS v9.4, whereas SPSS v26.0 will be used for statistical analysis. Chi-square and Fisher exact tests will be applied for categorical variables. Student’s *t* test will be used for continuous variables. The time-to-reoperation variable was evaluated using the Kaplan–Meier method, and the log-rank test was used to compare between the two groups. The two-sided *p*-value will be set to be significant at < 0.05.

## Results

**Fig. 2:**
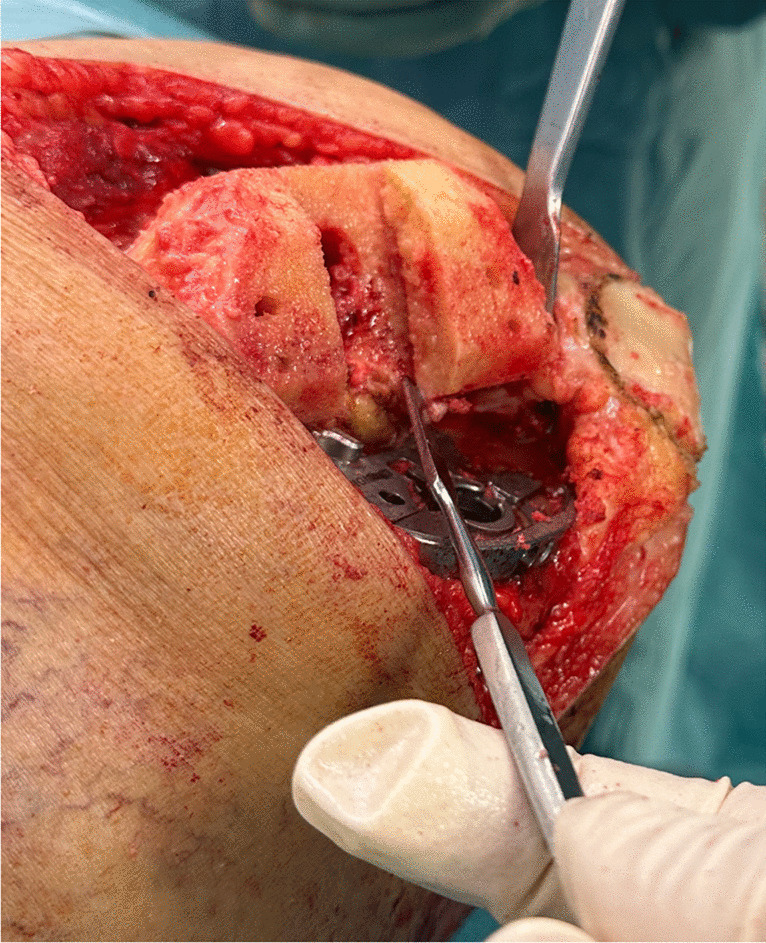
Groove or indentation in the undersurface of the reshaped lateral femoral condyle after saw cut in group 1 patients

**Fig. 3:**
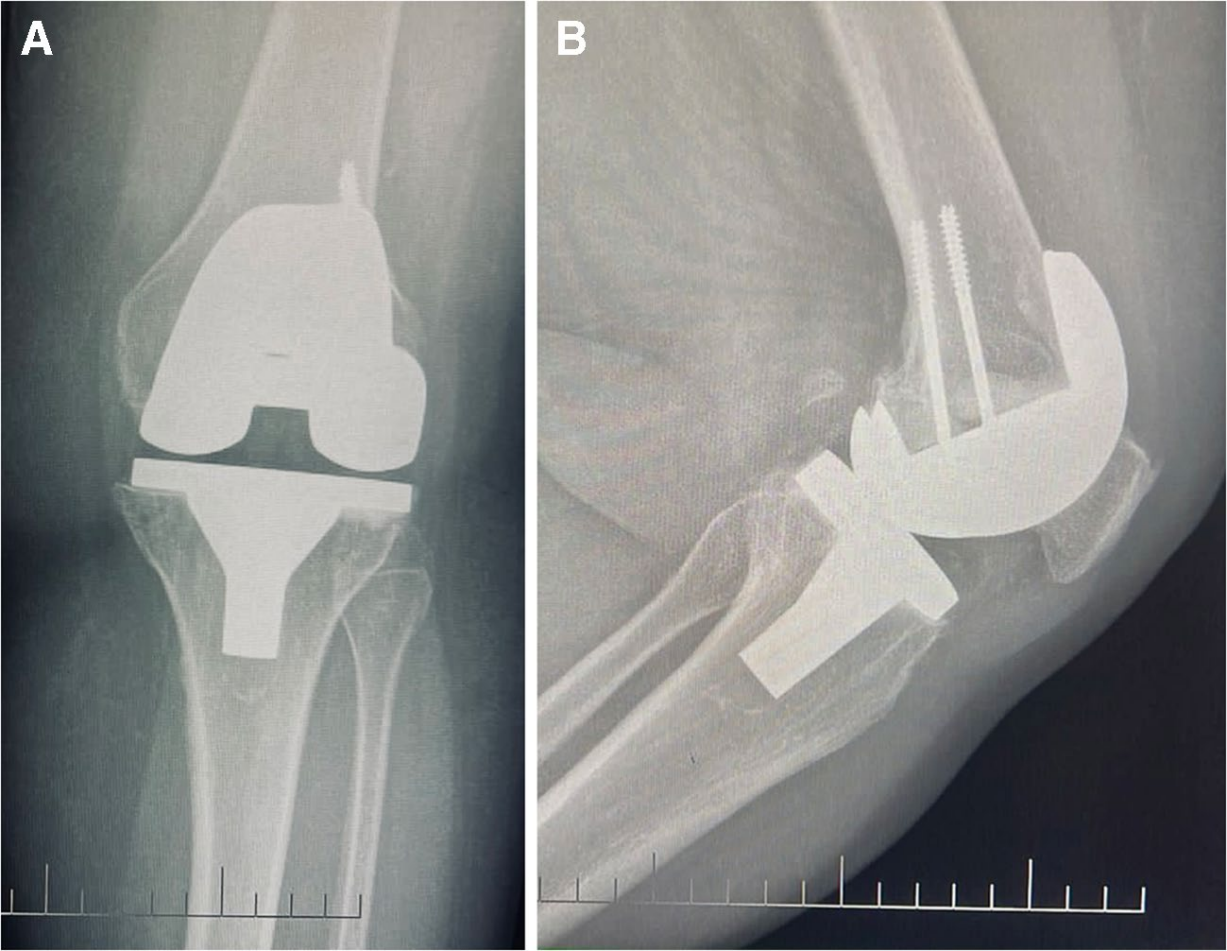
Four years postoperative X-ray of intraoperative lateral femoral condyle fracture fixed by two screws showing healed fracture

Regarding the operative data of our series, there was no difference in the operative time in both groups with an average of 78.2 min. The only observation that was seen in group 1 is the presence of mild groove or indentation in the undersurface of the reshaped femoral condyle (Fig. 2). It is probably created by the movement of the bone saw. It could be responsible for the only case of intraoperative lateral femoral condyle fracture in our series that was treated by two 4-mm cannulated screws and the femoral component was applied over it (Fig. 3).

The analysis of the registered knee society scores (Table 2) showed significant improvement from the preoperative knee scores. Comparing both groups showed significant differences between the two groups in the first KSS registered on six month post-surgery that revealed better results in group 2. The later KSS scores tended to equalize in the two groups till the last registered values.

**Table 2:** Comparison of the clinical outcome by knee society score in the two groups

Variable	Group 1	Group 2	*p*-value
Preop KSS	53.3 ± 7.1	51.7 ± 6.5	0.055
KSS (6 Mo postop)	89.5 ± 6.2	92.2 ± 4.3	< 0.001*
KSS (1 Yr postop)	91.5 ± 5.5	92.1 ± 6.7	0.626
KSS (last follow-up)	91.3 ± 6.3	91.8 ± 7.4	0.546

^*^Significant at *p* < 0.05

The reported complications of the series are shown in Table 3. There were no significant differences between the two groups except for the occurrence of periprosthetic fractures. There was a statistically significant difference between the two groups with a higher incidence of periprosthetic fractures in group 1. Revising this group of complicated cases showed that all the fractures affected females, osteoporosis was diagnosed in six out of these patients retrospectively, and all were of small femoral component sizes (Sigma sizes 2 and 2.5).

**Table 3:** Operative data and occurrence of complications in both groups

Variable	Group 1	Group 2	*p*-value
Operative time	78.1 ± 4.5, (62–85) min	79.2 ± 4.4, (65–87) min	0.534
Blood loss	211.4 ± 7.5, (130–300) mL	213.4 ± 6.8, (120–350) mL	0.367
Complications			
Superficial infection	2 (1.3%)	3 (2.5%)	0.795
Deep infection	2 (1.3%)	-	0.614
Intraoperative femoral fractures	2 (1.3%)	-	0.614
Distal femoral periprosthetic fractures	8 (5.3%)	1 (0.8%)	0.040*
Stiffness	1 (0.7%)	1 (0.8%)	1.000
Anterior knee pain	8 (5.3%)	3 (2.5%)	0.389
Loosening	2 (1.3%)	2 (1.6%)	1.000
Mid-flexion instability	3 (2.0%)	2 (1.6%)	1.000
Deep venous thrombosis	5 (3.3%)	5 (4.1%)	0.967

^*^Significant at *p* < 0.0

**Fig. 4:**
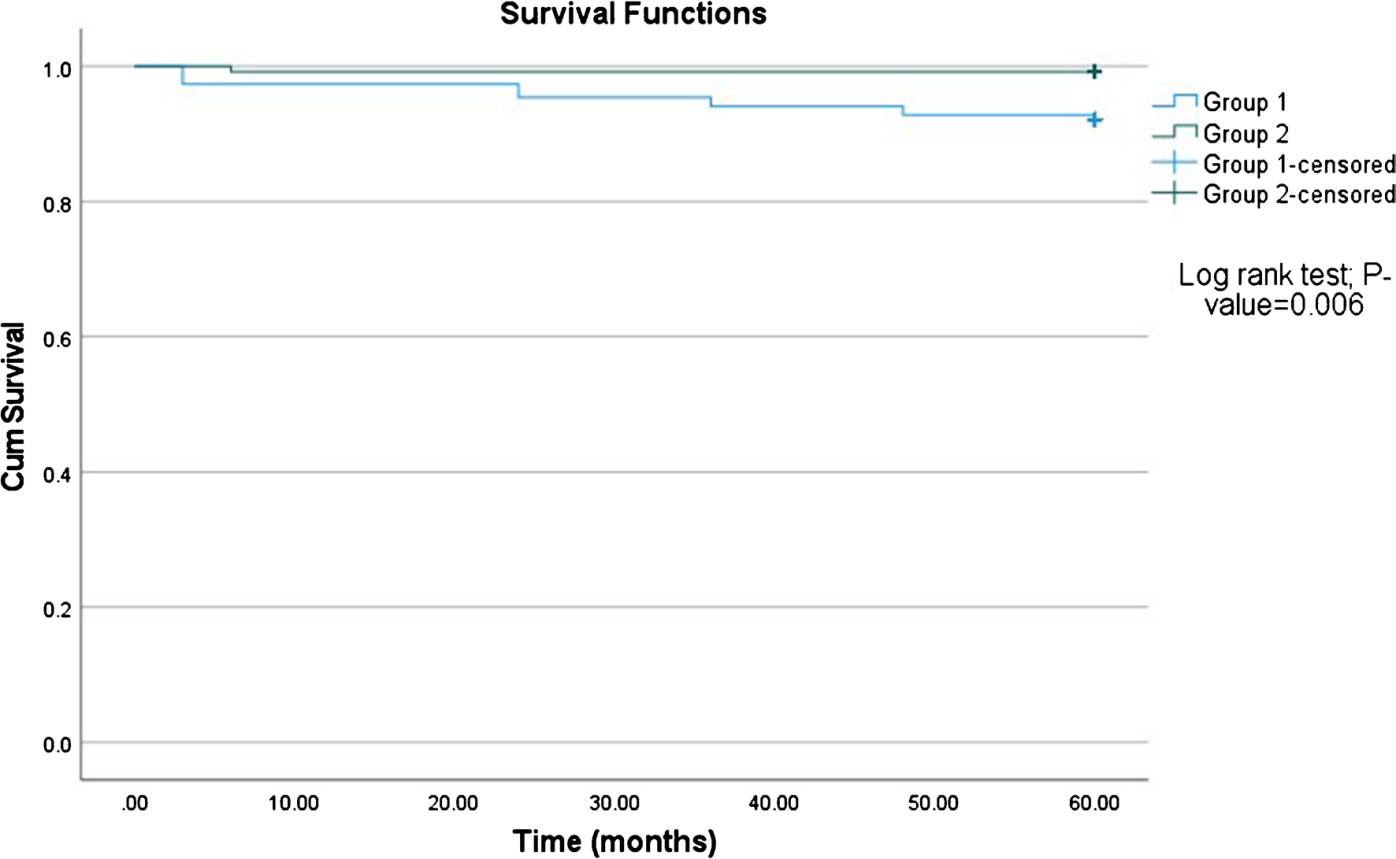
Survival analysis using the Kaplan–Meier method showing the prosthetic survival in both groups, *p* < 0.006

The survivorship analysis using the Kaplan–Meier method (Fig. 4) showed better outcomes in group 2. The time for reoperation was significantly shorter in group 1 than in group 2 as described by log-rank test, *p* < 0.006.

## Discussion

The total knee arthroplasty is a common procedure with a growing number of patients annually [2]. Refining and optimizing the surgical procedure will prove worthy on the long term. The ideal prosthesis is more bone-preserving to obtain stability and keep bone for future revisions without affecting the functional outcome. Despite the functional merits of PS implants, its major disadvantage of the PS implants is being less bone-preserving in comparison to CR implants [7]. Improving the prosthetic design and the surgical technique and instruments is mandatory to compensate for this deficiency.

Our series had compared two designs with different femoral box shapes and different box-cutting methods. The two designs are still in use in the market, and both are having long track records [14, 15]. This fact of being used for long gives an opportunity to identify long-term outcomes and obtain critical analysis to avoid further shortcomings [14, 16]. There was a good functional improvement that was achieved by both implants as shown on KSS of both groups. Those scores were comparable to many series [14, 16, 17]. There was a statistically significant difference in favour of the survival of the group 2 implants that depended on reamer-box creation. This advantage can be explained by the lower risk of periprosthetic fracture that was also statistically significant.

The higher incidence of the periprosthetic fracture in the saw-box cutting group 1 can be related to patient factors and implant factors. Patient factors include female gender in whom all the fractures occurred. The higher incidence in females was observed in several case series addressing the intraoperative and postoperative femoral fractures [18–20]. Another factor that needs to be highlighted preoperatively is the presence of concomitant osteoporosis which is more common in older post-menopausal females as well [20, 21]. An additional factor is the anatomically smaller femoral sizes [22]. Putting in mind that most periprosthetic fractures occurred in patient with smaller sizes of femoral component, this fact adds to the rationale and importance of preoperative templating for all patients undergoing TKA. Ettinger and his colleagues [23] concluded that 3D templating has a very high accuracy for the actual implant size prediction. Compared to this, 2D digital templating is an accurate method to approximately (± 1 size) determine the size of TKA components. We can advocate that the ideal patients for PS TKA are non-osteoporotic patients documented by a preoperative dual-energy X-ray absorptiometry (DEXA) scan, in addition to a larger size of distal femur in a preoperative template.

Implant factors include the size of the box cut relative to the distal femur [22]. In group 1, the size of the box cut is constant in all the range of sizes provided by the manufacturer and not related to the implant size. In addition, the shape of the box in group 1 is less bone stock preserving as it is not continuous from anterior to posterior. This may give an explanation to the higher incidence of fractures in small implant sizes 2 and 2.5. The volumetric analysis of the size of box cut of three different PS prostheses has shown that Sigma prosthesis has a larger box volume in all femoral sizes [7]. This problem is less evident in group 2 patients, in which the manufacturer made three variable box sizes that are related to the femoral component sizes. Also, the box is more bone-preserving with an anterior part of the femur is kept after the reaming for the box. Design of implants with smaller femoral boxes and narrower PE cam may offer a solution to address this challenge.

There was an important intraoperative finding of minor grooving or indentations after using the saw in group 1 (Fig. 2). It was reported that femoral condylar fracture can occur in PS TKA designs due to either the box cut itself or the stress riser created between the medial and lateral box corners and the metaphyseal cortices and can occur during the bone cuts, with insertion of implant trials, cementation of the final implant or postoperatively [24]. The lower KSS in the early six months postoperatively in group 1 can be attributed to the presence of minor cracks, occult fractures, or stress riser points that caused pain. We believe that surgical instruments to create different femoral cuts like bone saw should be more precise with appropriate speed and fewer non-intended oscillations and vibrations to prevent minor damages to the reshaped bones.

We can recommend that PS implant selection should stress on these points, how big is the box, is it related to the implant size, and how we are going to create it safely?

There are some limitations in our series: (1) The follow-up is short, although it is appropriate for the variables studied. (2) Other implant design factors may influence the outcome of the patients.

## Conclusion

The less bone-preserving PS designs can be optimized in order to reduce the risk of distal femoral perioperative fractures through:

1. Developing prosthetic designs focusing on creating smaller boxes that are relative to the femoral size.
2. Designing less-damaging instruments aiming to create a “more controlled box cut”
3. Proper selection of patients requiring attention to female osteoporotic patients and patients with small femora who may benefit from an alternative design without the need for a cam/post-PS mechanism.

## References

[1] Li JW, Ma YS, Xiao LK (2019). Postoperative pain management in total knee arthroplasty. Orthop Surg.

[2] Home | Agency for Healthcare Research and Quality, https://www.ahrq.gov/ (Accessed 2 June 2022)

[3] Kolisek FR, McGrath MS, Marker DR, Jessup N, Seyler TM, Mont MA, Lowry Barnes C (2009). Posterior-stabilized versus posterior cruciate ligament-retaining total knee arthroplasty. Iowa Orthop J.

[4] Siddiqi A, Levine BR, Springer BD (2022). Highlights of the 2021 American joint replacement registry annual report. Arthroplasty Today.

[5] 2018 annual report: fifth AJRR annual report on hip and knee arthroplasty data. American Joint Replacement Registry

[6] Nguyen LC, Lehil MS, Bozic KJ (2015). Trends in total knee arthroplasty implant utilization. J Arthroplasty.

[7] Graceffa A, Indelli PF, Basnett K, Marcucci M (2014). Analysis of differences in bone removal during femoral box osteotomy for primary total knee arthroplasty. Joints.

[8] Becher C, Heyse TJ, Kron N (2009). Posterior stabilized TKA reduce patellofemoral contact pressure compared with cruciate retaining TKA in vitro. Knee Surg Sports Traumatol Arthrosc.

[9] Puloski SK, McCalden RW, MacDonald SJ (2001). Tibial post wear in posterior stabilized total knee arthroplasty. An unrecognized source of polyethylene debris. J Bone Joint Surg Am.

[10] Indelli PF, Marcucci M, Pipino G (2014). The effects of femoral component design on the patellofemoral joint in a PS total knee arthroplasty. Arch Orthop Trauma Surg.

[11] Rodriguez-Merchan EC (2011). Instability following total knee arthroplasty. HSS J.

[12] Elkabbani M, Haidar F, Osman A, Adie Y, Dragos A, Tarabichi S (2022). Posterior stabilized total knee arthroplasty increases the risk of postoperative periprosthetic fractures. J Orthop Trauma Rehabilitation.

[13] Noble PC, Scuderi GR, Brekke AC, Sikorskii A, Benjamin JB, Lonner JH, Chadha P, Daylamani DA, Scott WN, Bourne RB (2012). Development of a new Knee Society scoring system. Clin Orthop Relat Res®.

[14] Victor J, Ghijselings S, Tajdar F, Van Damme G, Deprez P, Arnout N, Van Der Straeten C (2014). Total knee arthroplasty at 15–17 years: does implant design affect outcome?. Int Orthop.

[15] Hanusch B, Lou TN, Warriner G, Hui A, Gregg P (2010). Functional outcome of PFC sigma fixed and rotating-platform total knee arthroplasty. A prospective randomised controlled trial. Int Orthop.

[16] Kim YH, Park JW, Kim JS, Kulkarni SS, Kim YH (2014). Long-term clinical outcomes and survivorship of press-fit condylar sigma fixed-bearing and mobile-bearing total knee prostheses in the same patients. JBJS.

[17] Laskin RS (2001). The Genesis total knee prosthesis: a 10-year followup study. Clin Orthop Relat Res®.

[18] Agarwala S, Bajwa S, Vijayvargiya M (2019). Intra-operative fractures in primary total knee arthroplasty. J Clin Orthop Trauma.

[19] Lombardi AV, Mallory TH, Waterman RA, Eberle RW (1995). Intercondylar distal femoral fracture: an unreported complication of posterior-stabilized total knee arthroplasty. J Arthroplasty.

[20] Purudappa PP, Ramanan SP, Tripathy SK, Varatharaj S, Mounasamy V, Sambandam SN (2020). Intra-operative fractures in primary total knee arthroplasty-a systematic review. Knee Surg Relat Res.

[21] Ishii Y, Noguchi H, Sato J, Takayama S, Toyabe SI (2016). Preoperative bone mineral density and bone turnover in women before primary knee arthroplasty. Open J Orthop.

[22] Sherman WF, Mansour A, Sanchez FL, Wu VJ (2020). Increased intercondylar femoral box cut-to-femur size ratio during posterior-stabilized total knee arthroplasty increases risk for intraoperative fracture. Arthroplast Today.

[23] Ettinger M, Claassen L, Paes P, Calliess T (2016). 2D versus 3D templating in total knee arthroplasty. Knee.

[24] M Stefl RM Meneghini 2018 Posterior stabilized designs in modern total knee arthroplasty: Vestigial organs In Seminars in Arthroplasty 29 3 205 208 WB Saunders

